# Partial Splenic Embolization in a Patient with Hemophilia A and Severe Thrombocytopenia: A Case Report

**DOI:** 10.3390/hematolrep16020019

**Published:** 2024-03-26

**Authors:** Tomofumi Nakamura, Mitsuhiro Uchiba, Hirotomo Nakata, Takao Mizumoto, Toru Beppu, Shuzo Matsushita

**Affiliations:** 1 Department of Hematology, Rheumatology, and Infectious Diseases, Graduate School of Medical Sciences, Faculty of Life Sciences, Kumamoto University, Kumamoto 860-8556, Japan; 2 Department of Laboratory Medicine, Kumamoto University Hospital, Kumamoto 860-8556, Japan; 3 Department of Blood Transfusion and Cell Therapy, Kumamoto University Hospital, Kumamoto 860-8556, Japan; uchiba@kumamoto-u.ac.jp; 4 Department of Hematology, Rheumatology, and Infectious Diseases, Kumamoto University Hospital, Kumamoto 860-8556, Japan; nakatahi@gpo.kumamoto-u.ac.jp; 5 Department of Surgery, National Hospital Organization Kumamoto Medical Center, Kumamoto 860-0008, Japan; miztak@me.com; 6 Department of Surgery, Yamaga City Medical Center, Kumamoto 861-0501, Japan; tbeppu@yamaga-mc.jp; 7 Collaborative Research Program for Anti-Viral Agents and Hematological Diseases, Division of Clinical Retrovirology, Joint Research Center for Human Retrovirus Infection, Kumamoto University, Kumamoto 862-0973, Japan; shuzo@kumamoto-u.ac.jp

**Keywords:** hemophilia A, partial splenic embolization, thrombocytopenia, HIV, HBV, HCV

## Abstract

We report a patient with hemophilia A who underwent partial splenic embolization (PSE) for severe thrombocytopenia secondary to portal hypertension-induced splenomegaly, resulting in a stable long-term quality of life. The patient was diagnosed with hemophilia A and unfortunately contracted human immunodeficiency virus (HIV), hepatitis B virus (HBV), and hepatitis C virus (HCV) from blood products. He subsequently developed progressive splenomegaly due to portal hypertension from chronic HCV, resulting in severe thrombocytopenia. PSE was performed because he had occasional subcutaneous bleeding and needed to start interferon (IFN) and ribavirin (RBV) treatment for curing his HCV infection at that time. His platelet counts increased, and no serious adverse events were observed. Currently, he continues to receive outpatient treatment, regular factor VIII (FVIII) replacement therapy for hemophilia A, and antiretroviral therapy for HIV infection. Vascular embolization has been reported to be an effective and minimally invasive treatment for bleeding in hemophilia patients. PSE also provided him with a stable quality of life without the side effects of serious infections and thrombocytopenia relapses. We conclude that PSE is a promising therapeutic option for patients with hemophilia A.

## 1. Introduction

Hemophilia A (classical hemophilia) is an inherited bleeding disorder caused by a congenital deficiency or lack of coagulation factor VIII (FVIII) [1]. FVIII plays an important role in the coagulation process to control bleeding. Hemophilia A is an X-linked recessive genetic disorder that primarily affects males; however, female carriers of the gene may also be symptomatic [2]. In recent years, the development of several improved coagulation factor products has made it easier to control the coagulation process in patients with hemophilia A, and the quality of life for these patients has improved dramatically [3,4]. Nevertheless, hemostatic management of patients with hemophilia A who have traumatic bleeding injuries or undergo major surgery requires monitoring and adequate coagulation factor replacement. In addition, patients with hemophilia A need to receive care and support, including bleeding control and a recovery plan prepared by a medical team, including hematology specialists. The patient in this case study underwent partial splenic embolization (PSE), which has been developed as a minimally invasive treatment to avoid side effects such as severe abdominal pain, fevers, bacterial infections, and splenic abscesses.

Here, we present a male patient in his 40s who receives outpatient treatment for hemophilia A and human immunodeficiency virus (HIV) infection. He underwent PSE for severe thrombocytopenia caused by portal hypertension-induced splenomegaly due to chronic hepatitis C virus (HCV) infection when he was in his 20s [5]. To our knowledge, only one other partial case report of a patient with hemophilia A treated with PSE has been reported [6].

## 2. Case Report

**Figure 1:**
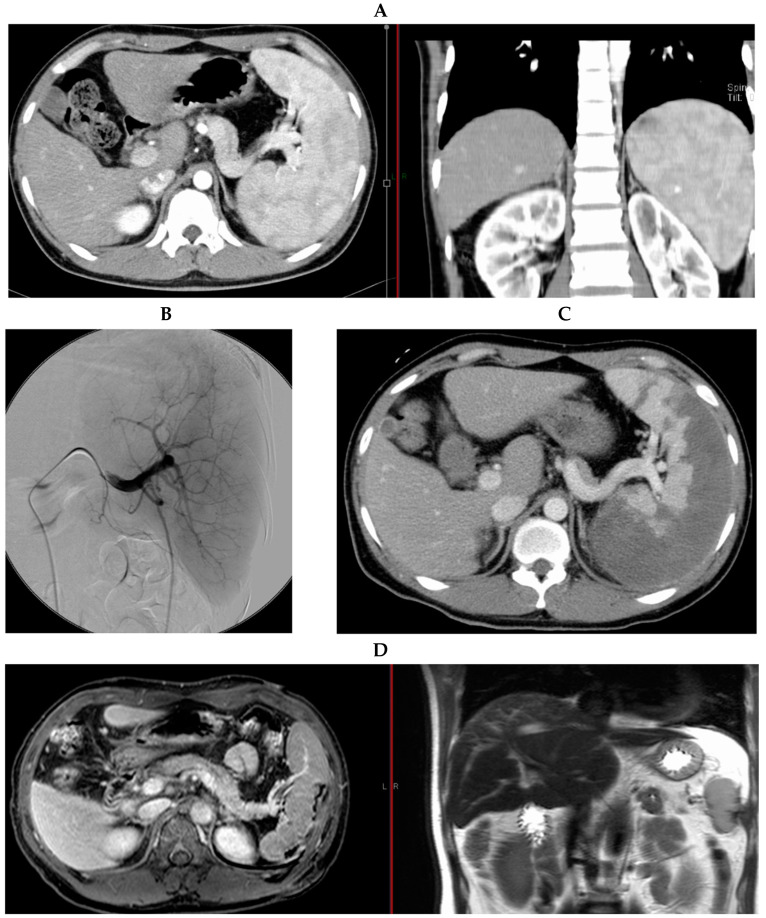
Imaging results for the patient. (**A**) Contrast-enhanced abdominal CT scan before PSE. The contrast-enhanced abdominal CT scan shows that the spleen is more than 12 cm in size at the coronal section, indicating splenomegaly. (**B**) Splenic angiography. Splenic angiography was performed to identify the embolized subregions of the spleen. (**C**) Spleen contrast-enhanced abdominal CT scan. The embolized areas of the spleen after PSE were confirmed with this contrast-enhanced CT scan. (**D**) Abdominal MRI conducted 5 years after PSE. The MRI was performed to confirm liver tumors 5 years after the PSE. There was no recurrence of splenomegaly.

At present, a male patient in his 40s is an outpatient at our hospital for the treatment of hemophilia A with efraloctocog alfa (Eloctate^®^, Biogen, Cambridge, MA, USA), and HIV infection with maraviroc (Celsentri^®^, Tokyo, Japan), lamivudine (Epivir^®^, ViiV Healthcare, Brentford, UK), and darunavir/cobicistat (Prezcobix^®^, Johnson & Johnson Innovative Medicine, Beerse, Belgium). When he was a child, his hemophilia A was classified as severe. He has received several types of recombinant FVIII replacement therapy for more than 20 years, depending on his bleeding symptoms and FVIII trough and peak levels. Now, efraloctocog alfa (30 IU/kg, two or three times per week) is being administered, resulting in the estimated 72 h trough and peak values of 2.6% and 71%, respectively. FVIII inhibitors are never detected. He unfortunately contracted hepatitis B (HBV), HCV, and HIV infections from blood products in the 1980s. He had severe thrombocytopenia that progressed (Table 1); occasional subcutaneous bleeding occurred despite adequate FVIII replacement therapy. Splenomegaly (Figure 1A) secondary to portal hypertension from chronic HCV was suspected as the cause of his severe thrombocytopenia and subcutaneous bleeding. Additionally, he needed to be treated with interferon (IFN) and ribavirin (RBV) to cure his HCV infection at that time.

When the patient was in his 20s, considering the risk of bleeding and neutralizing anti-FVIII antibodies induced by the high-dose administration of recombinant FVIII through invasive splenectomy, he underwent PSE in our hospital for management of his thrombocytopenia and subcutaneous bleeding, and initiation of IFN and RBV treatment for curing his HCV infection. No secondary abnormal findings such as esophageal/gastric varices secondary to portal hypertension were found on preoperative examination. A catheter was inserted into the femoral artery, and angiography was performed to visualize the splenic artery and segment for PSE (Figure 1B). Embolization of the splenic artery was performed, and a gelatin sponge was placed to slow the blood flow to prevent acute side effects.

**Figure 2:**
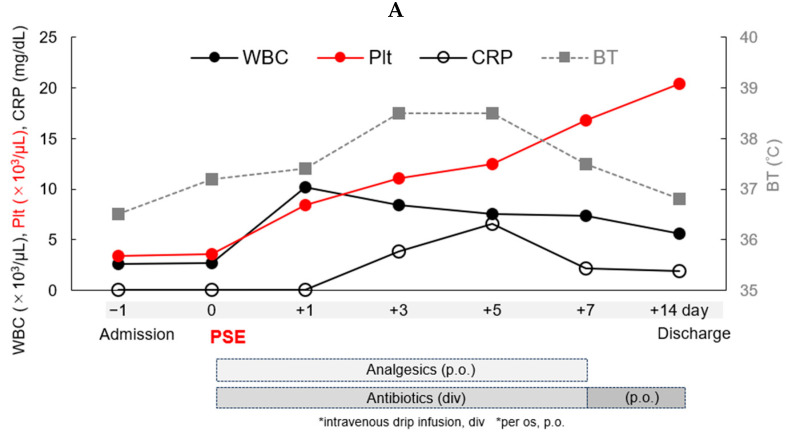

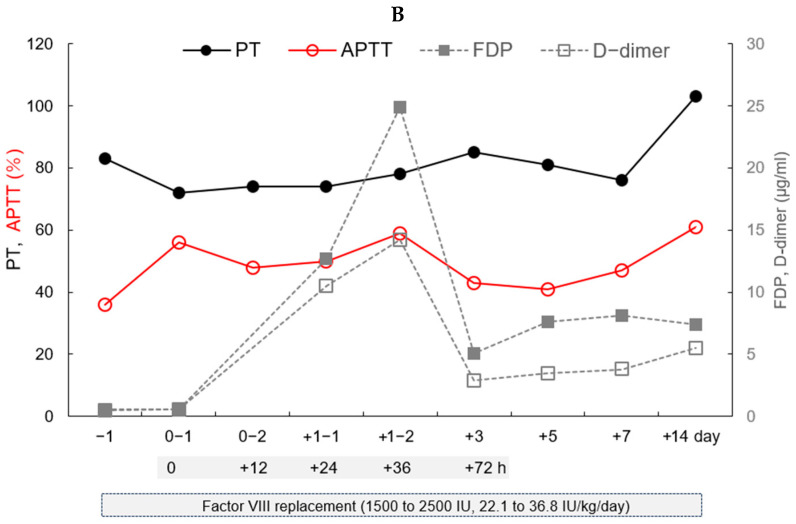
Clinical course of the patient during hospitalization. (**A**) Time course of the patient’s white blood cell (WBC), platelet (Plt), C-reactive protein (CRP), and body temperature (BT) levels during hospitalization. The time course of the laboratory data is shown for the WBCs in closed circles and a line, for the Plts in red circles and a line, for the CRP in open circles and line, and for the BT in gray squares and a dotted line, all during hospitalization. Analgesics and antibiotics were administered for one and two weeks, respectively, after PSE. (**B**) Coagulation status of the patient using the prothrombin time (PT), activated partial thrombin time (APTT), fibrin degradation products (FDPs), and D-dimer levels during hospitalization. The time course of the laboratory data is shown for the PT in closed circles and a line, for the APTT in red open circles and a line, for the FDPs in gray squares and a dotted line, and for the D-dimer levels in open squares and a dotted line, all during hospitalization. Adequate FVIII replacement was continued to maintain the APTT between 40 and 60% for 2 weeks during hospitalization.

After the procedure, contrast-enhanced computerized tomography (CT) confirmed that the splenic embolization area was approximately 90% (Figure 1C). His clinical course during hospitalization is shown in Figure 2. Administration of antibiotics and analgesics was begun post PSE, and left hypochondriac pain and a fever of approximately 38 °C appeared post PSE from Day 0. After Day 7, his pain, fever, and laboratory data gradually improved (Figure 2A). To achieve adequate hemostasis and per hemophilia management guidelines [7], 1500 to 2500 IU (22.1 to 36.8 IU/kg) of recombinant FVIII, Recombinate, was administered daily during hospitalization. His coagulation status was readily monitored through the activated partial thromboplastin time (APTT) instead of through FVIII activity because we did not see FVIII activity immediately, and other data such as the thrombin time (PT), fibrinogen degradation products (FDPs), and D-dimer levels were also monitored during hospitalization (Figure 2B). Post PSE, his platelet count increased from 4.0 × 10^4^ on Day 0 to 2.0 × 10^5^ /µL on Day 14. FDP and D-dimer levels were highest 36 h post PSE at 24.9 and 14.2 μg/mL, respectively, and decreased by 72 h. Slightly elevated levels of FDPs and D-dimer continued until Day 14. The patient showed no signs of inadequate splenic artery embolization, acute infection, or bleeding from the embolized spleen. The patient was discharged from the hospital in a stable condition on Day 14. No neutralizing anti-FVIII antibodies induced by the higher dose of continuous recombinant FVIII replacement were detected after the PSE.

Several months later, the patient started IFN and RBV treatment for his HCV infection and subsequently achieved a sustained virologic response. Magnetic resonance imaging (MRI) performed 5 years post PSE (Figure 1D) showed no liver tumor, and the spleen was normal in size. Currently, the patient receives regular treatments at our hospital for his hemophilia A and HIV infection and has experienced no serious side effects or deterioration in his quality of life.

## 3. Discussion

The patient’s HIV infection has been under control since the introduction of highly active antiretroviral therapy (HAART). However, we suspected that he had splenomegaly due to chronic HCV infection but without a cirrhotic pattern of fibrosis. The main cause of portal hypertension is cirrhosis of the liver [8], which accounts for approximately 80% of cases. Some cases of cirrhosis caused by HBV and HCV can be controlled with antiviral drugs. Peg-IFN and RBV treatment for curing his HCV also increased his sustained virologic response (SVR). Unfortunately, his splenomegaly progressed, leading to thrombocytopenia and subcutaneous bleeding despite adequate FVIII replacement therapy. Therefore, we opted for the minimally invasive treatment of PSE for his splenomegaly instead of a splenectomy.

PSE has attracted attention as a minimally invasive treatment to improve hypersplenism caused by portal hypertension; it may also preserve some of the important splenic functions compared to total splenectomy. This method was first reported by Maddison [9] in 1973 as total splenic embolization in patients with ruptured esophageal varices who were difficult to treat with other conservative methods. In 1979, Spigos et al. [10] reported on the safety of partial splenic embolization combined with prophylactic administration of antibiotics, which resulted in a sharp decrease in serious complications. PSE was considered to be a safe and reliable treatment method suitable for complications related to portal hypertension-induced splenomegaly. Moreover, a technique called the Takatsuka method [11] has also been reported, in which an estimated embolization rate can be calculated by embolizing the spleen in each of five subregions; with this method, thrombosis of the splenic terminal artery can be delayed until embolization by using an expandable metal coil as an embolization material. In 2014, Shimizu et al. reported that this method of PSE enabled the induction of regular-dose IFN therapy in patients without hemophilia A who had HCV cirrhosis [12]. Risk factors or therapeutic factors of PSE for thrombocytopenia in patients with cirrhosis have also been reported [13].

Patients with hemophilia in the era of inadequate factor VIII replacement therapy have problems with hemarthrosis, and vascular embolization of the joints in these patients has been frequently reported. As shown in Table 2, effective and minimally invasive treatments for patients with hemophilia also include vascular embolization for acute bleeding (spontaneous, postoperative, and traumatic), preoperative embolization for pseudotumors, and the efficacy of prostatic artery embolization for enlarged prostate [14,15,16,17,18,19,20,21,22].

When monitoring the coagulation status of the patient receiving FVIII replacement therapy after this PSE, the FDP and D-dimer levels decreased by 72 h without serious conditions such as disseminated intravascular coagulation. The patient showed no evidence of acute bacterial infection, bleeding from the necrotic spleen, or neutralizing anti-FVIII antibodies. These results suggest that patients with hemophilia A can safely undergo PSE with adequate FVIII replacement and administration of antibiotics.

In 2002, the British Committee for Standards in Haematology reported the uptake of guidelines for the prevention and treatment of infections in patients with an absent or dysfunctional spleen [23]. However, vaccines against encapsulated organisms such as pneumococcus, *Haemophilus influenzae*, and meningococcus were rarely administered in one survey of post-splenectomy patients in Japan [24], where guidelines existed only for splenectomies in patients with immune thrombocytopenia at the time. Our case study’s patient has not been vaccinated. His spleen is of a normal size, there are no Howell–Jolly bodies in his peripheral blood to suggest asplenia or hyposplenia [25], and his CD4 T cell count is normal, but we consider that these vaccines should be recommended.

## 4. Conclusions

Here, we first report in detail the general condition and coagulation status of a patient with hemophilia A who underwent PSE. PSE should be a useful minimally invasive treatment for patients with hemophilia for the management of complications associated with splenomegaly or thrombocytopenia.

## Figures and Tables

**Table 1:** Patient laboratory data at pre and post PSE.

Complete Blood Count				Coagulation				Biochemistry			
	Pre PSE	Post PSE			Pre PSE	Post PSE			Pre PSE	Post PSE	
WBCs	2600	3800	/μL	PT	12.7	10.9	sec	TP	8.5	6.8	g/dL
(White Blood Cell Count)				(Prothrombin Time)				(Total Protein)			
Hb	16.3	15.7	g/dL	PT	94	150	%	T-Bil	0.7	0.6	mg/dL
(Hemoglobin)								(Total Bilirubin)			
Plts	4.1	17.7	× 10^4^/μL	PT-INR	1.04	0.78		D-Bil	0.2	-	mg/dL
(Platelets)				(PT International Normalized Ratio)				(Direct Bilirubin)			
				APTT	60.5	41.3	sec	γ-GTP	149	62	U/L
				(Activated Partial Thrombin Time)				(γ-Glutamyltransferase)			
**Special Items**				APTT	36	46	%	AST	38	16	U/L
								(Aspartateamino Transferase)			
	**Pre PSE**	**Post PSE**		D-dimer	0.5	< 0.3		ALT	51	13	U/L
								(Alanine Aminotransferase)			
ICG/R15	20.4	-	%	FiB	197	-	mg/dL	CHE	137	235	U/L
(Indocyanine Green Retention15)				(Fibrinogen)				(Cholinesterase)			
ICGK	0.117	-		HPT	93	-	%	ALP	295	230	U/L
(ICG Krem)				(Hepaplastin Test)				(Alkaline Phosphatase)			
HIV-1 RNA	<50	N.D.	copy/mL	AT-Ⅲ	92	98	%	LD	178	139	U/L
(Human Immunodeficiency Virus RNA)				(Antithrombin-III Activity)				(Lactate Dehydrogenase)			
HCV RNA	85	N.D.	IU/mL	PLG	64	-	%	BUN	8.9	13.2	mg/dL
(Hepatitis C Virus RNA)				(Plasminogen)				(Blood Urea Nitrogen)			
HBsAg	0	-	mIU/mL	FDP-E	0.6	-	μg/mL	Cre	0.53	0.66	mg/dL
(Hepatitis B Surface Antigen)				(Fibrin Degradation Product E Fraction)				(Creatinine)			
HBsAb	159.3	-	mIU/mL	ProC	90	132	%	UA	6	6.5	mg/dL
(Hepatitis B Surface Antibody)				(Protein C)				(Uric Acid)			
				ProS	90	104	%	CRP	0.05	0.2	mg/dL
				(Protein S)				(C-Reactive Protein)			
				TAT	0.25	-	ng/mL	NH_3_	128	-	μg/dL
				(Thrombin Antithrombin Complex)				(Ammonia)			
				PIC	0.49	-	μg/mL	FBS	98	98	mg/dL
				(alpha2 Plasmin Inhibitor Plasmin Complex)				(Fasting Blood Sugar)			
				vWF	90	85	%	Haic	4.2	5.3	%
				(von Willebrand Factor)				(Hemoglobin A1c)			
				FⅧ	22	32	%	PIVKA-Ⅱ (Protein Induced by Vitamin K Absence or Antagonist-II)	17	-	mAU/mL
				(Factor Ⅷ)							
								AFP	5	-	ng/mL
								(Alpha Fetoprotein)			

N.D.: no detection; -: not tested. The data shown in blue and red indicate data below and above normal ranges, respectively. The post-PSE data represent 6 months after the PSE. The FⅧ data are from when he was an outpatient undergoing regular FⅧ therapy.

**Table 2:** Recent reports of vascular embolization in patients with hemophilia.

Group	Type	Year	Patients	Clinical Presentation	Ref.
Joints	Retrospective study	2023	24	Synovial hyperemia	[14]
	Systematic review	2020	15	Hemarthrosis	[15]
	Case series	2015	7	Hemarthrosis	[16]
	Case series	2009	7	Hemarthrosis	[17]
	Case series	2005	23	Hemarthrosis	[18]
Others (spine, chest, abdomen, pelvis, etc.)	Systematic review	2023	41	Pseudotumors	[19]
	Case series	2023	2	Prostatic hyperplasia	[20]
	Case series	2017	5	Acute bleeding	[21]
	Case series	2016	10	Acute bleeding	[22]

## Data Availability

All data generated or analyzed during this study are included in this article. Further inquiries can be directed to the corresponding author.
